# Human Intelligence Analysis through Perception of AI in Teaching and Learning

**DOI:** 10.1155/2022/9160727

**Published:** 2022-06-11

**Authors:** Pravin R. Kshirsagar, D. B. V. Jagannadham, Hamed Alqahtani, Quadri Noorulhasan Naveed, Saiful Islam, M. Thangamani, Minilu Dejene

**Affiliations:** ^1^Department of Artificial Intelligence, G. H. Raisoni College of Engineering, Nagpur, Maharashtra 440016, India; ^2^Department of Electronics and Communication Engineering, Gayatri Vidya Parishad College of Engineering (A), Madhurawada, Visakhapatnam 530041, India; ^3^King Khalid University, College of Computer Science, Center of Artificial Intelligence, Unit of Cybersecurity, Abha, Saudi Arabia; ^4^College of Computer Science, King Khalid University, Abha 61413, Saudi Arabia; ^5^Civil Engineering Department, College of Engineering, King Khalid University, Abha, Asir 61421, Saudi Arabia; ^6^Department of Information Technology, Kongu Engineering College, Perundurai, Tamil Nadu, India; ^7^Department of Biotechnology, College of Biological and Chemical Engineering, Addis Ababa Science and Technology University, Addis Ababa, Ethiopia

## Abstract

Instructional practices have undergone a drastic change as a result of the development of new educational technology. Artificial intelligence (AI) as a teaching and learning technology will be examined in this theoretical review study. To enhance the quality of teaching and learning, the use of artificial intelligence approaches is being studied. Artificial intelligence integration in educational institutions has been addressed, though. Students' assistance, teaching, learning, and administration are also addressed in the discussion of students' adoption of artificial intelligence. Artificial intelligence has the potential to revolutionize our social interactions and generate new teaching and learning methods that may be evaluated in a variety of contexts. New educational technology can help students and teachers better accomplish and manage their educational objectives. Artificial intelligence algorithms are used in a hybrid teaching mode in this work to examine students' attributes and introduce predictions of future learning success. The teaching process may be carried out in a more efficient manner using the hybrid mode. Educators and scientists alike will benefit from artificial intelligence algorithms that may be used to extract useful information from the vast amounts of data collected on human behavior.

## 1. Introduction

Human intelligence and the development of new technology are intrinsically linked to a college degree. Consequently, new possibilities and concerns for teaching and learning education are presented by advances in artificial intelligence; in addition, artificial intelligence has the potential to make significant changes to the basic design of higher education institutions [1]. Since Aristotle, philosophers have been unable to agree on a single definition of artificial intelligence. In the 1950s, scientists began looking for artificial intelligence answers to common problems in the world. Turing came up with the first answer to the question of when a system may be termed intelligent [2]. Human listeners will be able to recognize the difference between conversations with machines and those with other humans if the system is able to detect it, and if it does not, we will confess to having artificial intelligence (AI). It was later in 1956 that John McCarthy came up with the most comprehensive definition of artificial intelligence [3]: “AI is based on the premise... the intelligence that a machine or a programme shows; every component of learning or any other quality.”

Since the beginning of time, people have been learning and growing. Many factors go into determining how well kids and instructors are doing in school. Teachers often assess their pupils based on a set of predetermined criteria, such as their ability to maintain order, demonstrate innovation, work together with their peers, and demonstrate loyalty to the instructor [4]. First and foremost on the list is the student's ability to pay attention to and duplicate what they have learned in the classroom during the test. For a variety of reasons, marks/grades based on a student's understanding of the subject matter are at the top of the list [5]. As a teacher, you should be able to evaluate a student's performance by looking at their answers to questions over a certain period of time.

The instructor, who is already swamped with personally and professionally obligations, will have to put in more time and effort if they want to collect additional data. Preconceived ideas are used by most instructors in order for an evaluation of their other abilities to be as simple as possible. It is impossible to apply genuine skills, knowledge, and abilities of an assessed pupil. Students judge a teacher on a variety of qualities, such as their ability to engage with them on a personal level, as well as their subject matter expertise and degree of empathy [6]. Some professors have long been accused of being prejudiced towards some pupils because of their preference for children to be their favorites. When a kid is unable to grip the instructor, this is what occurs. Both the instructor and the student's attention are diverted in this setting [1].

Many of these variances lead to changes in learning outcomes and instructor teaching approaches. Students require an easy approach to study and are also encouraged to be mentors in the present situation [7]. Teachers' and teachers' materials were restructured due to this shift in organization. Artificial Intelligence (AI) has gained attention for the following reasons, among others:

Automation: Teachers can spend more time connecting with students by outsourcing basic processes like assessing, categorizing, and scheduling digital items.

Acclimation: The scholastic and corporate environments of today are not complete without the use of current technologies [8]. A recent Pew Research study found that 95% of young people regularly use their smart phones, with 45% of those individuals reporting that they are nearly constantly connected to the internet. Students may kick off the next wave of technical innovation with the aid of AI in the curriculum.

Integration: A managed IoT network and other IT projects, such as intelligent technology, may be used in conjunction with AI technologies to help educator's better serve kids.

Delineation: AI-driven analytics in education helps identify significant trends, draw critical markers, and assist instructors in developing the most successful curriculum that supports technological change by ensuring that information offered by instructors is useful and relevant.

Identification: Data analysis enables us to see how flexible AI systems will be able to pinpoint critical areas of learning for students [9]. You can notice and deal with the formation issue if you have strong protection and security systems.

Artificial intelligence (AI) is increasingly being used in schools around the country. With a two- to multiple deployment time, artificial intelligence (AI) and appropriate learning technology are emphasised in the 2018 Horizon report. It is predicted that AI in academia would grow by 42 percent during 2018 and 2022, according to Horizons Study's 2019 Higher Education Edition. However, the study also predicts that AI systems tied to learning and teaching will grow even more rapidly in the future a nonprofit Internet education [10]. Artificial Intelligence (AI) will have a huge impact on the future of postsecondary learning, according to Interface North.

Studies on the potential educational applications of artificial intelligence date back over three decades. This year will mark the AIED Society's 20th annual conference, which will be conducted in conjunction with the publication of the AI in Education magazine. There is still much to learn about the educational potential of AI technologies to assist students at all stages of their educational journeys [11]. Creating AI systems in postsecondary learning focuses on the ethical and legal concerns, notwithstanding the potential for AI to improve the quality of teaching. In the face of tight budgets, administrators may be tempted to deploy AI to replace teachers. You are not alone if you are concerned about losing your academic position to an expert system, chatbot, or other intelligent teacher. AI has the potential to enhance learning analytics, but on the other hand, such systems need vast amounts of data, particularly private data about students and instructors, which creates major privacy and security problems.

## 2. Objective

Learner planning process is the educator's examination and evaluation of numerous elements that influence learning, which serves as a foundation for establishing instructional techniques and enhancing the content of instruction. It is possible to analyze learning data in many ways in order to better understand how students learn, identify flaws in the process, and offer appropriate feedback. It is possible to use the findings of a learning scenario analysis to offer instructors a scientific foundation for implementing personalized instruction and resource recommendations over the course of a lesson, resulting in more precise and personalized instruction.

## 3. Review of the Literature

Baker and Smith [2] showed that AI may be broadly defined as “Computer systems that execute cognitive abilities, normally involved in human brains, such as learning and problem-solving.” There is no one technology that falls under the umbrella of artificial intelligence, they say. Many advanced devices and methodologies fall under the overarching category of machine learning. These include neural networks and algorithms. Artificial Intelligence (AI) and machine learning are often referenced together.

Popenici and Kerr [1] believed that identification and characterization, such as predicting if a student is likely to decline out of a program or be accepted into a programme, may be done using machine learning, which is an AI approach for categorization and profiling. Artificial Intelligence's area of machine learning comprises software that can detect trends and identify forecasts while also applying newly learned patterns to circumstances that were not originally planned.

Hicham et al. [3] introduced an analytical hierarchical technique for assessing teachers' performance, which included both quantitative and qualitative analysis, to make the recommendations. Examining if the credential comparability design is appropriate by maintaining neutrality in subjective assessment by different social environments and colleges and whether it is feasible to achieve this objective. Correlating evaluations with overall results is the gap in research that has to be bridged.

Singh and Pal [5] stated that learners' performance is analyzed using a variety of machine learning approaches. PCA, SVM, LDA, RNC, and ET are five machine learning algorithms used to categorise students' predictions. As far as these various methods go, SVM comes out on top with 94% accuracy. LDA ranks second with a precision of 93.21 percent. They were able to predict student's performance with the greatest degree of accuracy obtainable in the literature. In contrast to a separate training, the machine-learning technique incorporates the first-stage prediction as a feature to decrease generation mistakes and gain more information.

Kshirsgar et al. [4] developed diverse approaches that used classification techniques and actual dataset, relevant in all developing areas, and expand on artificial hybrid intelligence and standardization methods for classification and prediction of varied sets of data with high precision [6]. In cyber security, mobile computing, and cloud computing, the algorithms employed in various research projects are helpful for more accurate results with varied assessment criteria.

Gil et al. [7] believed that education systems have a daunting problem when it comes to predicting the reasons of student's dropout. Consequently, they investigated if data mining techniques would aid in resolving this problem at every school. In this case, the data mining classification approach was able to accurately forecast student dropout rates. The most widely utilised data mining algorithms based on C4.5 and Naive Bayes were employed to find the student's dropout indicators. Tenfold cross-validation was used to train and evaluate these two separate categorization methods. Educators are notified to take necessary measures to help students improve their performance via specialised training and counselling.

Hasan et al. [8] showed that student's performance was predicted using a data set of 1170 students. 89.74% accuracy was obtained using Decision Tree Classifier using Linear Discriminant Analysis, Gradient Boosting, Random Forest and SVC classifiers, and KNN and KNN classifiers.

Nurakhmetov [10] stated that ML techniques include supervised, unsupervised, reinforcement, and deep learning. It is the most often used ML approach that uses labelled sets of input-output data to train the system to predict the outcome for fresh unlabeled input. Unlabeled data is used to train the system for prediction in unsupervised learning. Rather than using data to train the system, reward learning makes use of the consequences the system gets from its own activities. Deep learning techniques, which are the most difficult to implement, use numerous layers of representation to uncover hidden patterns in massive datasets. It is possible for the model to learn and adjust its internal parameters by using this machine learning approach. There are several applications for machine learning (ML) algorithms that create enormous volumes of data, including e-commerce, consumer items, biomedical imaging, and supply chains.

## 4. Technological Structure of AI

Intelligence teaching may take several forms, including information processing, imaginative learning environments, and information predictive analysis. AI-enabled education is becoming more important [12]. An intelligent system of education is one that delivers timely and individualized training as well as assessment to both teachers and students. As a result, they use a wide range of computer technologies, including machine learning [13] and cognitive learning theory, to enhance learning value and efficiency.

**Figure 1:**
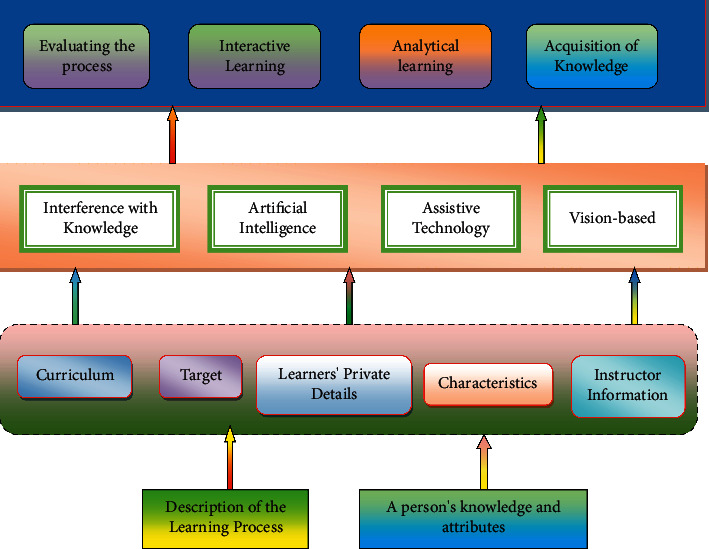
Technological structure of AI.

A variety of strategies are used in an AI system for learning assessment, suggestion, active learning, and data gathering based on machine learning [14]. Artificial Intelligence educational standards are often separated into two parts: the system (which includes learner model, teaching model, and knowledge model) and the intelligent technology [15].  Figure 1 shows how important it is to use models to create a data map in order to develop structures and rules of association for the data gathered on education.

The model serves as the brain of an AI system, which is powered by many technologies. The learner model is crucial in AI learning systems because it helps students become more self-sufficient in their own learning. It is based on data gathered from students throughout the learning process. A student's capacity to learn is evaluated by looking at how they think and what they can do. Then, learners' information mastery is traced using information evaluation. Learning outcomes are linked to a variety of elements, such as learning materials, resources, and instructional practices, via the use of “learner modeling.” The most prevalent types of learning include expert knowledge, guidelines for making mistakes that learners often make, and misconceptions. This educational paradigm, which integrates the information space model with the learner model, allows instructors to make adjustments to their teaching methods and practices [16]. There is a noticeable correlation with student's behaviour as education progresses. Artificial intelligence (AI) could always use the built-in teaching principles of the coaching paradigm. The output of a user interface is an explanation of a learner's performance using a variety of different input mediums. In addition to natural language interaction, voice recognition, and student mood detection, the enhanced human-machine interface delivers AI-related capabilities.

### 4.1. State-of-the-Art and Future Prospects

**Figure 2:**
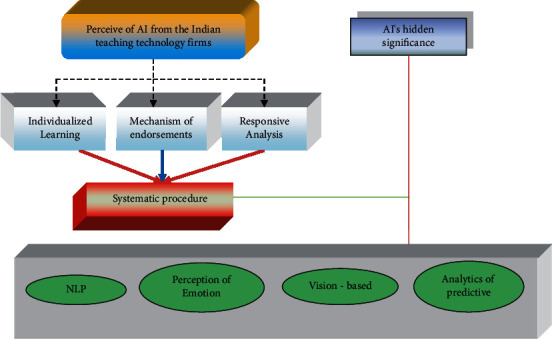
Artificial intelligence in the Indian education system: state-of-the-art and future prospects.

The key results of the research are shown in Figure 2. Personalization of learning is the most common application of AI in education, according to all responders from the Edtech companies. Students' educational outcomes depend heavily on the work of their instructors. Teachers are finding it more challenging to maintain a consistent emphasis on each student's growth in larger classes [17]. In addition, children learn in various ways and at varying speeds. There are many different ways to learn, and a one-size-fits-all strategy may not work for everyone. One of the participants noted:

“To guarantee that every learner has an exceptional instructor who tailors the whole learning experience, we use a sophisticated technological platform.”

Rule-based algorithms used by education technology firms may be used to identify a student's learning path and give tailored learning material. Many of these companies feature large databases with millions of questions, extensive coverage of ideas, animated movies, gamified quizzes and flashcards, and a variety of other educational tools [18]. The following is an explanation provided by one of our readers: “each topic is rated according to its difficulty level by the instructor. A fast quiz results in an immediate answer, and students are given motivational remedial ways to help them improve their understanding of the material. Because of this, students have an easier time picking up new skills and retain more information. In addition, students may take chapter exams or practice questions on a subject of their choosing thanks to the personalization mechanism [13]. It selects relevant questions from a database of millions of questions and allows the learner to practice.” “AI assists in picking the correct practice question from your collection of questions,” one responder said in an interview. As a result, subject matter experts and data scientists work together to personalize information.

The AI system makes suggestions based on a person's strengths and limitations. The customization engine and the recommender systems overlap since suggestions are tailored to a student's specific issue areas. Remedial films, practice problems based on the incorrect answers, and advice to refer to certain portions of the textbook are some of the suggested remedial materials. In addition, the number of questions a student needs to answer to fully comprehend a given idea may be customized using these technological platforms. There is not a set of questions for each idea in advance [19]. To determine if a learner needs more practice with comparable problems or should go on to a more challenging level, the algorithm analyses their responses. As a result, individualized suggestions try to increase the student's comprehension of the issue area that is most relevant to them.

### 4.2. An Intelligent Approach to AI for Education and Training

Our Human Intelligence (HI), how it works best with AI, how it complements AI, and what new information and skills we need to learn are all critical questions that must be answered immediately. In order to distinguish between the skills and information that will be essential in our future AI-enhanced society and those that we no longer need to focus on in our education and training systems, we must take this crucial step [20]. In the present scenario, we have developed incredibly complex AI that can learn, and we have also developed AI that can assist us in developing even more powerful HI. It is impossible to overestimate AI's contribution to education and knowledge advancement [21]. Knowledge of the world as opposed to academic knowledge is what we mean when we talk about social knowledge. In Luckin's definition of academic intelligence, this is multi- and transdisciplinary knowledge and comprehension. It is difficult to separate skills from knowledge, and this relationship must be understood [22]. Knowledge is only useful to learners if they have the abilities to apply it, and knowledge is only useful to learners if they have the skills to apply it. Separating the ways in which AI may benefit students and instructors is, nevertheless, an important consideration in educational contexts.

**Figure 3:**
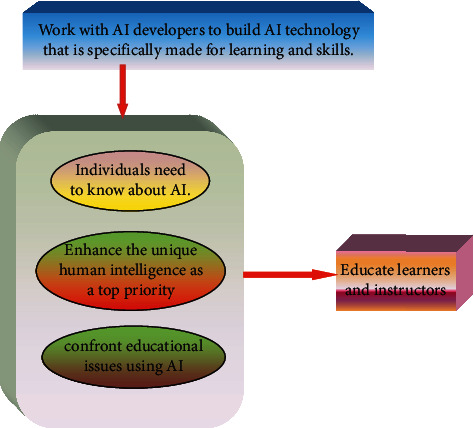
An intelligent approach to AI in education and training.

Figure 3 shows an intelligent approach to AI in education and training.

## 5. The Hybrid Teaching Mode

**Figure 4:**
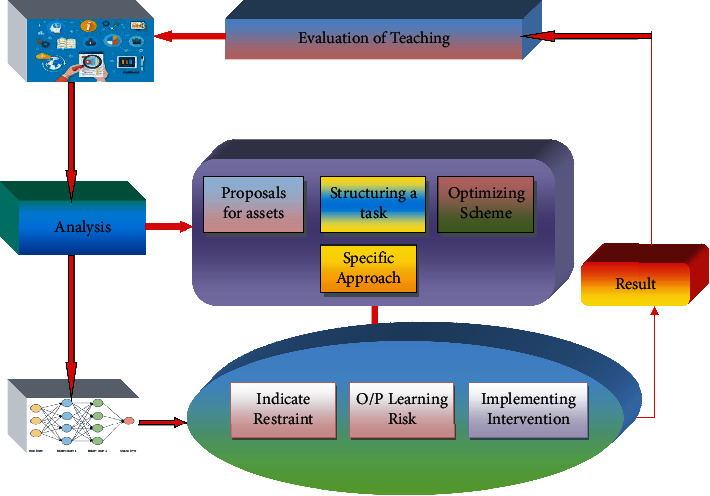
Design of hybrid model for teaching and learning using AI.

Figure 4 illustrates the suggested hybrid teaching method. First, the data is collected from the learning environment, and then the artificially intelligent algorithms are used to create the models and finally the results are used to carry out the validation of teaching. Using data gathered from online learning and academic practice, the suggested method is predicted to enhance teaching and learning outcomes [23]. Algorithms play a vital role in teaching mode, and the grouping analysis and ANN algorithm are utilised to identify the distinctions between students' knowledge sources and learning styles. We will go through each aspect of the hybrid teaching approach in more depth below: data preprocessing, model creation, and assessment. In the first step, the data is collected, cleaned, and transformed into the proper forms from the online network by preparing the data [24]. Students' names, course completion rates, forum postings, assignment completion rates, course engagement time, and the number of interactive days in the course are all included in the data, which is gleaned straight from the online platform. During preprocessing, erroneous or unnecessary data is weeded out and discarded, such as removing data that is missing or inconsistent, as well as those that are not relevant to the research. In addition, the data are smoothed or constructed into approximate forms through data transformation, which removes noise from the data [25]. To minimize the amount of range characteristics, flattening may also use binning, regression, and clustering approaches. The mining process was aided by the creation of new attributes and the addition of new attributes from the supplied list of attributes.

## 6. Methodology

### 6.1. Artificial Neural Network

**Figure 5:**
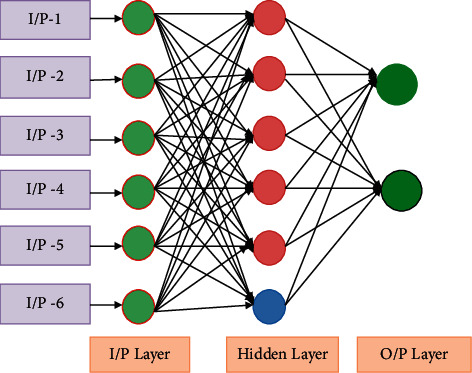
Architecture of artificial neural network.

Input, one or more hidden layers, and output layers make up the ANN's frame. Neurons in each layer of the network form a collection of interconnected modules. These neurons communicate with each other through a weight-based system of interconnections. The following layer's neurons are all related to the previous layer's neurons. In the neural network, the data are shown in the input layer. The output layer will display the neural network's output based on the input data. Hidden layers allow these networks to compute causal relationships between input and output.  Figure 5 depicts the artificial neural network's architecture [26].

There should be a balance between the number of concealed layers and their significance. For the majority of issues, a single hidden layer is often required for investigation. Experimentation will determine how many hidden layer neurons to use, starting with the smallest possible number and gradually increasing as the problem size increases. The neural network operation is supplied with a set of input and target output values. Input parameters will be chosen to influence output parameters. Many genetic difficulties may be modeled using the back-propagation approach [17].

Network error models can be reduced by using back-propagation algorithm, which is in essence a difference-reduction method:

(1) $$\begin{array}{c} E=\overset{K}{\underset{J=1}{\sum }}\overset{n}{\underset{i=1}{\sum }}\left(e_{i}\left(j\right)-t_{i}\left(j\right)\right). \end{array}$$

*e*_*i*_(j) and *t*_*i*_(j) are the estimated and targeted values, respectively. There are “*n*” output nodes and “*k*” training samples in this example.

The ANN primary examples with a randomization weights. Depending on how many mistakes are replicated, the weights are adjusted.

(2) $$\begin{array}{c} \Delta W_{ij}\left(n\right)=\propto \frac{dE}{dW_{ij}}+\eta \Delta W_{ij}\left(n-1\right). \end{array}$$

When *n* is more than one, we have a weight rise between the *i* and *j* nodes in adjacent iterations, which is the training rate and impetus. To train a recurrent neural network effectively, it is necessary to conduct a thorough evaluation and adjust the learning rate accordingly. Some prior ANN models on nutrient removal in a biological therapy were referenced in various papers.

**Figure 6:**
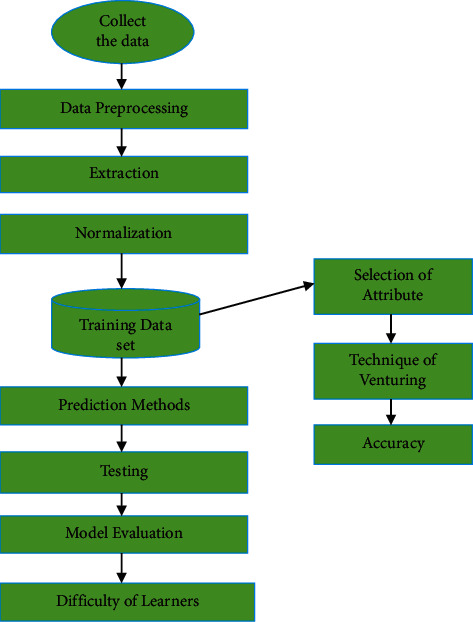
Flow diagram of performance of ANN.

The number of predictor variables employed by the machine learning algorithms is reduced as a result of this preprocessing phase. Among other things, it simplifies machine learning models, reduces computation time of models, enhances their generalisation capabilities, prevents overtraining, and reduces the number of resources necessary to estimate the difficulty of a student in a session. Predictive models' accuracy may be improved by combing through a large number of potential combinations of characteristics [27]. Using data from previous student logs, these classifiers are able to forecast the difficulty of the forthcoming session for the student and the instructor. By knowing how challenging a student's next class will be, teachers may prepare accordingly. Training and testing datasets for the classifiers comprised students' exercise-related attributes and their mean grades from the same sessions.  Figure 6 shows the flow diagram of performance of ANN.

## 7. Result and Discussion

As part of the suggested hybrid teaching mode, educators actively plan and develop teaching activities, speak openly with students, accomplish instructional tasks in the midst of a collision of thought, and so on, In the classroom, teachers may use the findings of artificial intelligence algorithms to assist them improve their curriculum, devise activities, and change their teaching tactics. Using artificial intelligence algorithms, customized resources, and personalized coaching for teachers, the hybrid teaching mode develops a teaching instructional framework tailored to each individual student.

**Figure 7:**
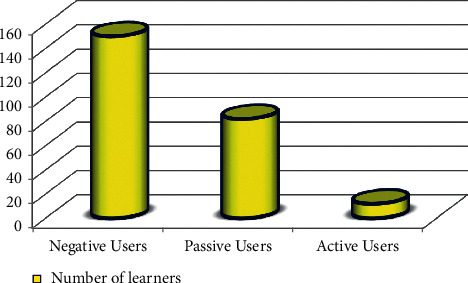
Analysis of learners.

240 students were sorted into three groups once the data was prepared. The students are divided into three groups based on how they respond to the parameters: active, passive, and negative. More than 62% of the course material has been learned by active users, who participate actively in learning activities, talk in the forum, and complete their assignments. Traditional learning approaches, such as watching videos, browsing course materials, and doing assignments, are exclusively used by passive learners. Negative learners engage in passive learning, engage in little or no learning activities, and lack the capacity to learn on their own without the help of others. For example, there are only 12 active and 82 passive members in Table 1 and Figure 7, respectively; the remaining 150 members are negative members.

### 7.1. Teaching and Learning Supported by AI

**Figure 8:**
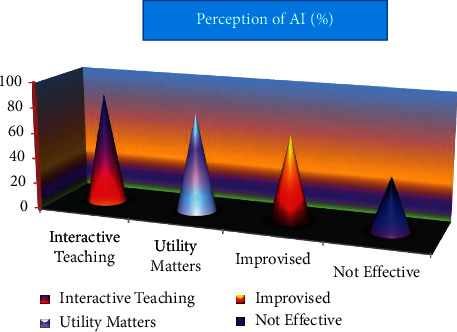
Teaching And Learning supported by AI.

Only a minority of instructors are hesitant to embrace new technologies. What if the current generation of students had the same experience as the previous generation, and if it was a pleasant one, why not the current generation of students? The coded bits reveal that although the majority of instructors believe that AI represents the future, there are a handful who see it as a threat to their profession. An equal number of participants said that AI aids in learning and urged that AI be used in a limited manner.  Table 2 and Figure 8 reveal that most instructors believed that only a small fraction of participants would be successful if they were assisted by AI in their teaching and learning.

### 7.2. Performance of ANN

**Figure 9:**
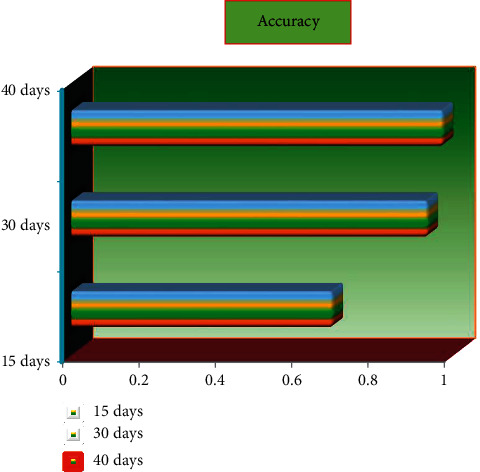
Accuracy.

We can observe from the table below that ANN has three different prediction accuracies: 0.789% accuracy in the first two weeks, 0.932% accuracy in the first five weeks, and 0.978% accuracy in the first eight weeks. This shows that SVM successfully predicts performance using data from the first five weeks, and we can deduce that the type 1 error is substantially smaller than type 2 errors. Analyzing online data, we offer relevant resources for students with different learning styles and optimize the teaching technique; we provide early warning, personal assistance, and intervention based on the findings. The students all passed the course as a consequence of the hybrid teaching method, which is a good sign.  Figure 9 and Table 3 show the accuracy.

(3) $$\begin{array}{c} \text{Type}-1\text{ error}=\frac{\text{FP}}{\text{FP}+\text{TN}},\\ \text{Type}-2\text{ error}=\frac{\text{FN}}{\text{TP}+\text{FN}}. \end{array}$$

## 8. Conclusion

Many studies show that the usage of artificial intelligence in many aspects of our daily life is on the rise, and this trend seems to be consistent. When it comes to schooling, according to the findings of this research, educators and students alike should learn more about how artificial intelligence (AI) might help them improve their educational abilities. It was also shown that the best application of AI technology may lead to improved outcomes. It was predicted that AI education will become more accessible in the future via a variety of platforms or trends. Learning management systems, in general, may keep track of how students are interacting with the material they are studying. As a result of diverse learning styles, learners' behaviour activities and performance will vary. Algorithms provide educators with empirical information that may be used to enhance learning and teaching. As early as the fifth week, results from real-world applications show that the hybrid teaching method reliably predicts students' future performance. By proposing teaching materials, planning teaching activities, and implementing teaching interventions, all students complete the course.

## Figures and Tables

**Table 1:** Analysis of learners.

S. no.	Types of learners	Number of learners
1	Negative users	150
2	Passive users	82
3	Active users	12

**Table 2:** Teaching and Learning supported by AI.

S. no.	Teaching and learning pattern	Perception of AI (%)
1	Interactive teaching	87
2	Utility matters	78
3	Improvised	67
4	Not effective	42

**Table 3:** Accuracy.

S. no.	Data	Accuracy	Type-1 error	Type-2 error
1	15 days	0.789	0.005	0.123
2	30 days	0.932	0.004	0.078
3	40 days	0.978	0.003	0.089

## Data Availability

The datasets used and/or analyzed during the current study are available from the corresponding author upon reasonable request.
